# Metabolic Engineering of Microalgal Based Biofuel Production: Prospects and Challenges

**DOI:** 10.3389/fmicb.2016.00432

**Published:** 2016-03-31

**Authors:** Chiranjib Banerjee, Kashyap K. Dubey, Pratyoosh Shukla

**Affiliations:** ^1^Department of Environmental Science and Engineering, Indian School of MinesDhanbad, India; ^2^Department of Biotechnology, University Institute of Engineering and Technology, Maharshi Dayanand UniversityRohtak, India; ^3^Enzyme Technology and Protein Bioinformatics Laboratory, Department of Microbiology, Maharshi Dayanand UniversityRohtak, India

**Keywords:** biofuel, microalgae, *Chlamydomonas reinhardtii*, metabolic engineering, bioreactor

## Abstract

The current scenario in renewable energy is focused on development of alternate and sustainable energy sources, amongst which microalgae stands as one of the promising feedstock for biofuel production. It is well known that microalgae generate much larger amounts of biofuels in a shorter time than other sources based on plant seeds. However, the greatest challenge in a transition to algae-based biofuel production is the various other complications involved in microalgal cultivation, its harvesting, concentration, drying and lipid extraction. Several green microalgae accumulate lipids, especially triacylglycerols (TAGs), which are main precursors in the production of lipid. The various aspects on metabolic pathway analysis of an oleaginous microalgae i.e., *Chlamydomonas reinhardtii* have elucidated some novel metabolically important genes and this enhances the lipid production in this microalgae. Adding to it, various other aspects in metabolic engineering using OptFlux and effectual bioprocess design also gives an interactive snapshot of enhancing lipid production which ultimately improvises the oil yield. This article reviews the current status of microalgal based technologies for biofuel production, bioreactor process design, flux analysis and it also provides various strategies to increase lipids accumulation via metabolic engineering.

## Introduction

In recent times, microalgae have gained attention due to the depletion of non-renewable fossil fuel. Biofuel produced from microalgae has benefit to reduce 78% emission of carbon dioxide, 98% decline in sulfur emissions and 50% decline of particulate matter after combustion (Brown and Zeiler, 1993). Microalgae are now realized excellent source for biofuel compared to other traditional sources of energy viz., hydro, wind, or from other biomass such as plants, household and industrial waste. Microalgae are having an extra advantage to be used as alternate source i.e., fixation of large amount of CO_2_(100 tons of microalgal biomass fixes 183 tons of CO_2_ Chisti, 2008). Biomass produced from microalgae has excellent prospects to convert into biofuel due to the low emission of CO_2_ compared to other biomass sources. Bioconversion methods which comprises (i) fermentation of the microalgae biomass to produce ethanol and hydrogen; (ii) extraction of oils from the microalgae for biodiesel production (Skjanes et al., 2007) which is a biodegradable, renewable, eco-friendly fuel.

Microalgae based biofuel can be obtained after transesterification reaction. In transesterification reaction acid/alkali catalyst was used (Fukuda et al., 2001) and lipids were converted using methanol or ethanol into ethyl/methyl esters of fatty acids (Xuan et al., 2009). Completion of reaction two phases were generated the heavy phase (crude glycerine + excess alcohol + water+ impurities). Light phase was centrifuged and dehumidified, which results in biodiesel that should have characteristics which matched the ASTM standards (Maa and Hanna, 1999). Worldwide microalgae base biofuel has been gaining interest to be blend in CI engines (Sgroi et al., 2005).

Metabolic engineering approach coinciding with other strategies like genetic engineering, flux balance, identifying target pathway and its enzymes are the key factors toward the achieving the target of producing fuel from microalgae. In order to produce biofuel from microalgae, an effective biochemical pathway should be constructed with a proper selection of host and other prerequisite parameters like pathway targeting and it's modeling toward desired product formation. As the metabolic pathway are very complex in nature, the difficulties lies in marking an appropriate pathway capable of producing biofuels.

The interest has been driven due to the genome sequences are available of more than 30 microalgae but metabolic pathway is still in the initial stage. Identification of genes involved in enzyme and integrating with complex metabolism is really difficult without base/model system. This difficulty has led to development of *Chlamydomonas reinhardtii* as a model system in eukaryotic microalgae. Genome Scale Metabolism Model (GSMM), Flux Balance Analysis (FBA) can be integrated with transcriptomics, proteomic data which is further constructed and analyzed after establishment of a base system.

The commercially viable biofuel recovery from microalgae is not realistic due to the (i) little biomass recovery (ii) cost of downstream processing and validation. Furthermore, the viability of microalgae based biofuel can be achieved only by the (i) designing advanced photobioreactors (ii) developing low cost technologies for biomass harvesting, drying, and oil extraction (iii) development of biorefinery approach.

The overall production of such biofuel from microalgae can be enhanced by the genetic engineering approaches and adopting metabolic pathways engineering for augmented lipid production. Besides above techniques for improvement in the possibility to harnessing microalgae for biofuel a new emerging technologies i.e., biotic or algal-bacterial interactions for enhancement of microalgae growth and lipid production are also explored (Costa and Morais, 2011). Researchers have been focused on the development of high lipid content microalgae using metabolic engineering approach and cultivated in large scale open pond for biofuel production, and also capture carbon dioxide from coal-fired power plants as biological emission control process (Brennan and Owende, 2010). Now-a-days consumption of microorganisms and their metabolic products by human beings are one of the most significant fields, and possible due to the development the field of biochemical engineering.

Present review focuses mainly on the challenges encountered in the commercial production of microalgae based biofuels and the application of metabolic engineering approaches to overcome these difficulties.

## Lipid biochemistry in algae

Lipid biochemistry processes are very important in extracting fuel from microalgae. Identification and target to increase fatty acid content in microalgae different enzymes involves in rate limiting steps of pathways. Though the study related to fatty acid content is a forward step but still much more clarity is required. Therefore, biochemistry of lipid droplets is an important factor to be studied for enhancement of biofuel production.

Lipid biogenesis can be improved by identifying the important node and internode in its pathway. Importing single glucose transporter gene can divert the basic metabolism by replacing glucose in place of light (Zaslavskaia et al., 2001). Identifying tricky pathway through different flux analysis model and enzymes are important aspect and well-reviewed (Banerjee et al., 2016) viz. overexpressing DGAT gene in *Chalmydomonas reinhardtii* doesn't lead to increase its lipid content (La Russa et al., 2012) but again overexpressing the same gene in *Phaeodactylum tricornutum* resulted in increased lipid droplets by 35% (Niu et al., 2013).

**Figure 1:**
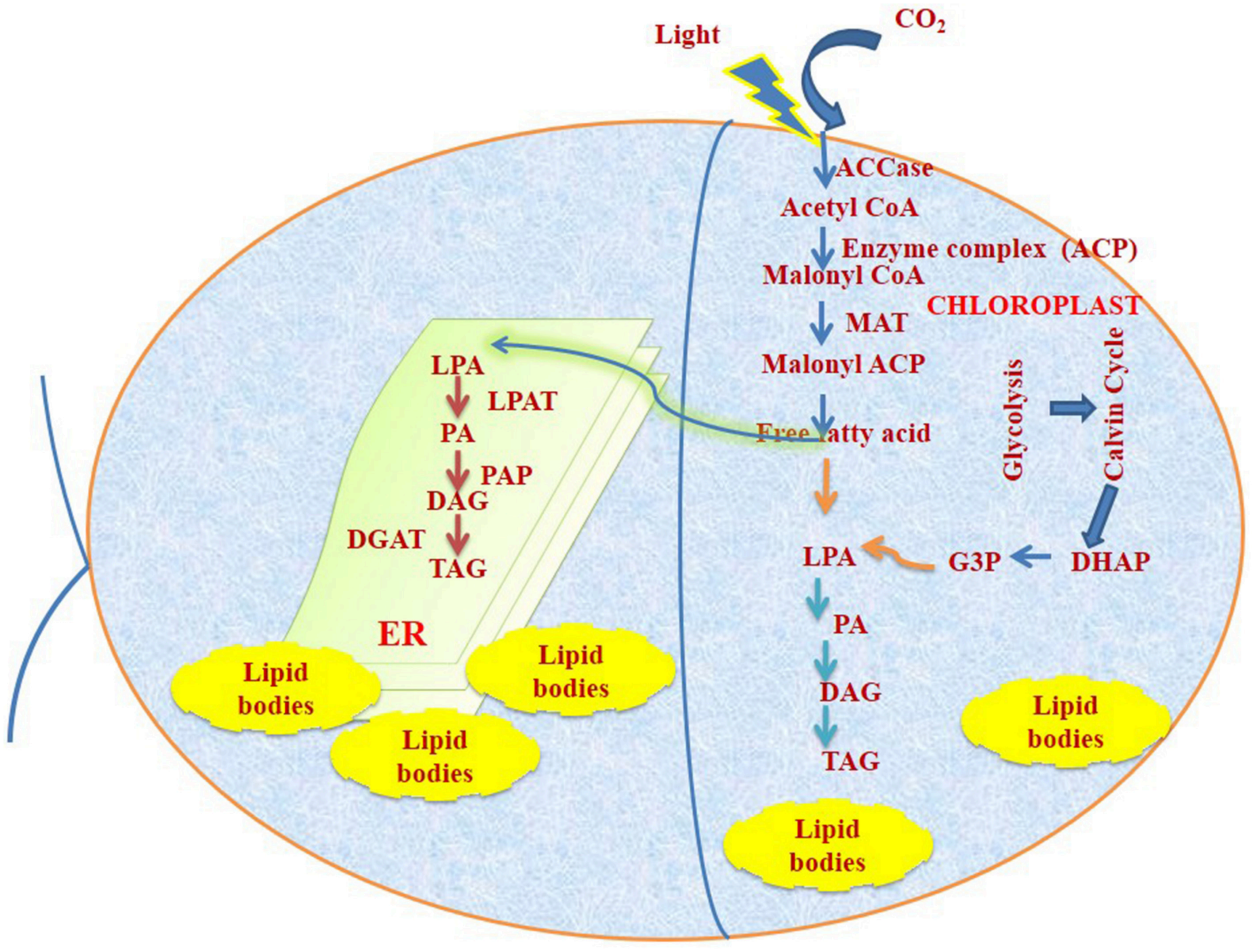
**Fundamental representation for TAG synthesis and accumulation pathway in *C reinhardtii***. DAG, diacylglycerol; DGAT,diacylglycerol acyl transferase; G-3-P, glycerol-3-phosphate;. ACCase, acetyl-CoA carboxylase; ACP, acyl carrier protein; FFA, free fatty acid; DHAP, dihydroxyacetone phosphate; MAT, malonyl-CoA:ACPtransacylase; PAT, lysophosphatidic acid acyltransferase; LPA,lysophosphatidic acid; PA, phosphatidic acid; PAP, phosphatidic acidphosphatase; TAG, triacylglycerol.

Two different key conserved enzymes namely, type-II fatty acid synthase (FAS) and Acetyl CoA Carboxylase (ACCase) are found to be linked with fatty acid synthesis pathway. These enzymes are present in chloroplast and ACCase is a rate limiting pathway for fatty acid biosynthesis. ACCase, carboxylate Acetyl CoA to form malonyl CoA and FAS elongate the fatty acid chain by two units (Post-Beittenmiller et al., 1991, 1992). The acetyl CoA pools will be fulfilled from glycolysis or from TCA Cycle. Diagrammatic representation of compartmentalization of fatty acid biosynthesis is represented in Figure 1.

TAG synthesis mainly occurs from two different routes:

### Kennedy pathway

Glycerol-3-phosphate is acylated followed by acylation of lysophosphatidic acid resulting in formation of phosphatidic acid. Phosphatidic acid gets dephosphorylated to produce diacylglycerol by diacylglycerol acyl transferase (DGAT) and then finally to triacylglycerol (TAG).

### Acyl CoA independent pathway

In this pathway acyl group is transferred from phospholipids by phospholipid:diacylglycerol acyltransferase (Hildebrand et al., 2013).

In *Chlamydomonas* sp. genes for DGAT was found more when compared to *Arabidopsis* which further compounds the complexity in microalgae. In plants, plastid becomes the house for fatty acid synthesis. The plant lipid (TAG) production is not restricted to specialized cells but in microalgae it can be triggered by stress. (a) specific lipid like betaine lipid which was not reported in plants (Mongrand et al., 1998; Klug and Benning, 2001).

Isoprenoid molecules are the key components for measuring biofuel from diatoms due to the prevalence of two different biosynthetic pathways for isoprene viz. methylerythritolphosphate (MEP) and mevalonate (MVA) pathway (Lohr et al., 2012).

## Metabolic engineering of lipid catabolism in microalgae

Lipid engineering in microalgae can be achieved by conventional, genetic engineering and metabolic engineering approaches.

### Conventional methods

Includes nutrient deprivation, physical stress like temperature, salt stress, and heavy metal stresses etc. which are thought to increase the activity of several enzymes. Among different types of stress especially nitrogen stress are being highly reported to trigger the TAG accumulation in different class of microalgae. Nitrogen, phosphorus stresses are being responsible for activating acyltransferase's and variation in phosphorus transporter system respectively, which again triggers TAG accumulation in microalgae (Khozin-Goldberg and Cohen, 2006; Dubey et al., 2015).

Though, temperature will vary depending on microalgae (Tamiya, 1957) but normally it has optimal growth rate at 15–26°C (Hu et al., 1998). Thus, in day time higher photosynthetic activity results in high growth rate and *vice versa* in night. Similarly in case of pH some can resist high pH owing to their higher adaptability. Higher CO_2_ means higher biomass but this will also decrease the pH (Kumar et al., 2010). The actual reason for increasing lipid in other stresses like pH, heavy metal is still unknown. Besides the reporting of high cell density culture, some recent biopolymeric harvesting approach has also been reported (Banerjee et al., 2012, 2013, 2014). Stresses can become the constructive strategy for increasing the lipid droplets due to the inherent advantages like ease in handling method, requirement of no skilled labor. On other side it also lowers down photosynthetic activity resulting in lower growth rate (Li et al., 2008). Nutrient limitation is a key player to increase lipid droplets and is widely reported. It is one of the expensive and easy scheme where redirecting of metabolic flow occurs toward lipid (TAG) formation. In this facet the major disadvantage are slow growth rate and low photosynthetic activity. Since lipid productivity is directly proportional to cell number therefore two stage cultivation approach may be employed to circumvent the above stated problem but photosynthetic one still remains.

Different approaches for increasing lipid biogenesis in microalgae have been represented in Table 1.

**Table 1:** **Different approaches for increasing lipid biogenesis in microalgae**.

**Algae used**	**Method applied**	**Nutrient conditioning**	**Outcome**	**References**
**CONVENTIONAL TECHNIQUES**				
*Nannochloropsis oceanica* IMET1	Steady state continues culture	high light intensity and nitrogen replete	Higher neutral lipid and biomass; 11% trehalose of Dry cell weight (DCW)	Xiao et al., 2015
*Chlorella sorokiniana*	Normal culture condition	Nitrogen was replete and/or depleted	Dynamic carbon partitioning between starch and lipid which lead to produce one of the compound in replete/deplete condition respectively	Li et al., 2015
*chlorella vulgaris var* L3	Normal culture condition	Nitrogen starvation	Fatty acid synthesis gene and Carbohydrate metabolism genes are unregulated resulting in increased lipid (TAG) content (2.7 times)	Ikaran et al., 2015
*Chlamydomonas reinhardtii*	Mixotrophic condition	Low light conditions	5–27% higher dry cell weight than Wild type(WT)	Zhou et al., 2015
*Chlorella zofingiensis*	Photoautotrophically grown	N-starvation	Lipid increases to 24.5% of dry weight	Zhu et al., 2014
*Neochloris oleoabundans*	Normal culture condition	Low light, high pH and nitrogen starvation		Santos et al., 2014
*Chlorella minutissima* UTEX 2341	Normal culture condition	NaCl, Fe^3+^ and nitrogen starvation	lipid content increase to 2.5 times	Cao et al., 2014
**Algae used**	**Gene/platform involved**	**Nutrient conditioning**	**Outcome**	**References**
**GENOME EDITING TOOLS**				
*Nannochloropsis oceanica*	overexpressing *NoD12* under the control of the stress inducible promoter	Nitrogen starvation	Increased long-chain polyunsaturated fatty acids and TAG production;	Kaye et al., 2015
*Synechocystis* sp. PCC6803	*bicA*	Atmospheric CO_2_	Grew almost twice growth rate and biomass with respect to wild type	Kamennaya et al., 2015
*Chlorella* sp.	Quadruple codon optimized gene construct for Kennedy pathway by electroporation	Normal culture medium	6% (wt) of TAG and 60% (wt) of total lipid content	Chien et al., 2015
*Phaeodactylum tricornutum*	*PtME* overexpressing	Nitrogen deprivation	Neutral lipid increases to 2.5-fold	Xue et al., 2015
*Nannochloropsis oceanica*	Overexpressing *NoD12* under the control of the stress-inducible promoter	nitrogen starvation	substantial increase in TAG (LC-PUFA)	Kaye et al., 2015
*Phaeodactylum tricornutum*	co-expressing otelo5 and otd6pt with biolistic approach	NA	Accumulation of high value omega-3 long fatty acid	Hamilton et al., 2014
**Algae used**	**Software/algorithm used**	**Flux involved Pathway**	**Outcome**	**References**
**MODELING/PLATFORMS FOR METABOLIC ENGINEERING**				
*Chlorella protothecoides*	Integrated flux balance	Calvin-Benson Cycle, glycolysis, PP pathway, the TCA cycle and the biosynthetic pathways leading to biomass	Detailed quarrying of metabolic functionality Optimizing Carbon fluxes in autotrophic and heterotrophic growth leading to lipid production	Wu et al., 2015
*Tisochrysis lutea*	Dynamic Reduction of Unbalanced Metabolism (DRUM)	Photosynthesis, Lipids, biomass synthesis	Lipids and carbohydrates accumulation and consumption	Baroukh et al., 2015
*Chlamydomonas reinhardtii*	metabolic flux analysis	Algal biomass enhancement	Modeling of *C. reinhardtii* growth and metabolism.	Kliphuis et al., 2012
*Synechocystis* sp. PCC 6803	Flux Balance Analysis	TCA cycle, an alleged glyoxylate shunt, and the role of photorespiration	Integration of TCA, Glyoxylate and respiration and reconstructing of metabolism (alternating diurnal light/dark) cycles	Knoop et al., 2013
*Phaeodactylum tricornutum*	Remodeling of metabolism through FBA	TCA cycle and Urea cycle	Uncovering the fluxes involve of carbon to lipids formation under nitrogen stress	Levitan et al., 2015
*Chlamydomonas reinhardtii*	FBA	Detailed biomass equation in all growth regimes	Primary metabolism which includes intracellular flux values for lucid engineering of *C. reinhardtii*.	Boyle and Morgan, 2009

### Metabolic approach

Metabolic engineering strategy is defined as tuning of metabolic pathways in a cell to trigger the target metabolite production. Achieving such targets various strategies can be adopted which are listed below:

- Flux balance analysis
- Improving photosynthetic efficiency (Increasing light penetration/Decreasing cell shading)
- Engineering different enzymes toward lipid biogenesis
- Identifying rate limiting enzymes/committed step
- Carbon partitioning/capture
- Mathematical modeling
- Over expression of a gene/multiple gene
- Transcription factor engineering

The following are the major favorable points toward production of lipid droplets in microalgae. Lipid biogenesis is governed by three steps namely Acetyl CoA carboxylation, Chain elongation followed by TAG formation. Furthermore, synthetic biology aspect requires preliminary information about the organism (microalgae). Whole genome sequencing of model as well non model microalgae is required in order to reconstruct the metabolism. Reconstruction of metabolic fluxes using stoichiometric model i.e., S.v = 0; Where v is a vector of fluxes and S is matrix, and matrix is constructed by balancing the masses in each of the cell compartment of *Chlamydomonas reinhardtii* (Boyle and Morgan, 2009).

Kyoto Encyclopedia of Genes and Genomes (KEGG; Ogata et al., 1999) and MetaCyc (Caspi et al., 2005) are the major key resource to trace the metabolic pathways. Gene expression dataset or differentially expressed genes can also be put into the picture to draw metabolic construction using pathExpress (Goffard and Weiller, 2007). Recently, fluxome study of *Pseudomonas fluorescens* (Lien et al., 2015) regarding fructose metabolism in EMP, EDP, PPP, TCA cycle has also been performed.

Nutrient limitation is a key player to increase lipid droplets and is widely reported. It is one of the expensive and easy scheme where redirecting of metabolic flow occurs toward lipid (TAG) formation. In this facet the major disadvantage are slow growth rate and low photosynthetic activity. Since lipid productivity is directly proportional to cell number therefore two stage cultivation approach may be employed to circumvent the above stated problem but photosynthetic one still remains. Metabolic flux analysis using GC-MS and LC-MS/MS under photoautotrophic growth in *Synechocystis* sp. PCC6803 has also been depicted to locate the carbon distribution using INST-MFA algorithms with high accuracy (Roesler et al., 1997; Young et al., 2011).

Cytosolic Acc ase was transferred to *Brassica napus* from *Arabidopsis* in order to increase the fatty acid content (Gu et al., 2011). Nevertheless, after transformation the fatty acid content increases to 6% which led to identification of some other limiting steps. Acc ase is present as a multi domain enzyme in most eukaryote and the heteromeric four different subunit from *Jatropha curcas* was characterized using g 5 RACE technique and was found maintain the conserved domain. A strain of *Escherichia coli* that yields anteiso-branched fatty acids to decrease the freezing point and escalate the fluidity (Haushalter et al., 2014). Analysis by qPCR was also done to evaluate the differential gene expression pattern which is directed toward Pyruvate and acetyl-coA synthesis under nitrogen depriving condition (Li et al., 2012). Similarly, metabolic engineering for Fatty acid synthase is also a challenging target due to its multi subunit structure and have a multipoint controls.

Current progress in whole genome sequencing and its annotation will definitely pave the way toward lipid biogenesis. Recent genetic tools like Multi gene approach, transcription factor like CRISPR/TALEN, reverse genetics are well reported. Manipulating genetic code will show a manipulation in metabolic pathway and its flux toward the target/desired compound. Though knockdown technology (RNAi), genome editing through modern tools have been described and is established in *Chlamydomonas reinhardtii* (Kim and Cerutti, 2009), *Dunaliella salina* (Jia et al., 2009). But still we are unable to establish a base line system where every microalgae can be manipulated. Recently, robust and nuclear expression of xylanase1 in *C reinhardtii* with viral 2A peptide has been achieved. This technology involves less number of transformation steps. High quality transcriptomic reads to the tune of 45% were assembled and identified in case of *D. tertiolecta* for ascertaining lipid genesis and carbohydrate metabolism network (Rismani-Yazdi et al., 2011). Knock down gene expression by two microRNAs in C *reinhardtii* for *RBCS1*/2 and *MAA7* gene was also reported (Zhao et al., 2009). Similarly overexpression of CrDGTT4 (type-2 diacylglycerol acyl-CoA acyltransferase) from *C. reinhardtii* with SQD2 (sulfoquinovosyldiacylglycerol synthase 2) as a promoter will also increase TAG accumulation under phosphorus starvation (Iwai et al., 2015).

## Biochemical engineering in microalgal biofuel (lipid production enhancement)

Advantages of microalgae for biofuel application over the other fuel crops have been thoroughly reviewed (Schenk et al., 2008) which includes short life cycles (1–10 days) than plants, possess higher light conversion rates, small area is needed for the production of the same amount of biomass as compared with traditional biofuel crops (Schenk et al., 2008; Greenwell et al., 2010).

Microalgae could be grow in pools, tanks and bioreactors which can be placed on waste land, deserts and areas which are not suitable for food production (Greenwell et al., 2010); eventhough it can also be grown in wastewater (Yun et al., 1997).

In recent days biochemical engineering has gaining interest to the industries and researchers is the cultivation, and harvesting of microalgae in continuous mode. The fatty acids produced through microalgae can be extracted and converted into biodiesel (Brown and Zeiler, 1993). Among microalgae species, oil contents can reach up to 80%, and levels of 20–50% are quite common (Powell and Hill, 2009). The microalga *Chlorella* has up to 50% lipids and *Botryococcus* has 80%. The commercial production of lipids from microalgae for biofuel production is based on open tanks and tubular bioreactor (Jimenez et al., 2003) which is common in Israel, Japan, Taiwan, Indonesia, United States and China.

## *In silico* metabolic engineering

Another approach of metabolic engineering could be generated by designing the large-scale models which use various *in silico* tools to decipher the role of different metabolites, genes, transcripts and crucial enzymes responsible for metabolic fluxes (Patil et al., 2004; Schmidt et al., 2010). There are enough reports which establish the role of different computational techniques which prove to be significant for understanding key components of lipid regulation and can be very crucial for researchers working in the area of biofuel.

However various other reports also summarizes various metabolic network modeling and flux balance analysis which plays a vital role while designing some novel pathways or establishing an idea about enhanced recovery of lipids from microalgae (Schuhmann et al., 2012). As a whole, the availability of metabolic models and *in silico* tactics on identifying key residues of lipid metabolism can be the role establishing characteristics and give quite sufficient information. Additionally, the improvement of the available models on transcriptomics, proteomics and metabolomics based data will facilitate to obtain key components toward good quality biofuel. Certainly, such information generated through *in silico* metabolic engineering on microalgal lipid metabolism has to be appraised by wet lab experiments.

## Challenges and conclusions

The metabolic engineering of microalgae is an significant area of research due to enormous interest on generating efficient biofuel. As algae has shown the highest divergence (67.7% distinct) so these findings regarding genes divergence will through a light toward a positive hope for lipid bio-genesis. A survey of literature above has established the idea that research has been carried out only in model microalgae (*C. reinhardtii*), so more focus has to be put on genome sequencing, and rigorous genome scale metabolic flux based analysis is required toward TAG accumulation in different lineages of microalgae. In our opinion, first we should target the key genes responsible for lipid biosynthesis. As we had genome sequenced for plant as well for algae the methods that are belong employed in plant can easily be replicated in microalgae also.

Certainly, the various type of stress responses on microalgae is a key tool for increasing the lipid droplets. Moreover, the accessibility of nutrients during stress condition will have a definite role in lipid productivity. It is well documented now that during stress conditions the microalgal metabolism will shift toward the storage of energy rich essential molecules in form of triacylglycerol are which could be efficient source of lipid. But definitely, writing these aspects in research article and to get it done practically is not impossible but it's tough in case of complex lipid biogenesis pathway.

## Author contributions

All authors listed, have made substantial, direct and intellectual contribution to the work, and approved it for publication.

### Conflict of interest statement

The authors declare that the research was conducted in the absence of any commercial or financial relationships that could be construed as a potential conflict of interest.
